# Prize Essay

**Published:** 1841-09

**Authors:** 


THE WESTERN JOURNAL.

Vol. IV.—No. III.

LOUISVILLE, SEPTEMBER 1, 1841.

PRIZE ESSAY.

It will be seen that the Prize Essay of Professor Yandell is inserted in this number, to the exclusion of our usual Review matter. And some of our esteemed correspondents must take this as an apology for the non-appearance of their communications. We shall present our usual variety in the next number, and will give insertion to some of the communications which have been delayed, though our limits will not admit of the great number that we have recently received.

The following is the report of the committee of the Medical Society of Tennessee, appointed to examine the essays on Bilious Fever.

"The committee, whose duty it was to award the prize to the successful essayist, beg leave to report, that they received four essays on Bilious Fever, for examination, and, after carefully examining them, unanimously award the prize to the writer whose essay bears the following motto: '*Sanos sospitare ægrosque sanare.*' The committee beg leave to deliver the essays over to the Society, with the sealed packet unbroken.

FELIX ROBERTSON.
JOHN H. ATKINSON.
THOS. R. JENNINGS.

NASHVILLE, May 3, 1841.

"The Secretary being ordered to break the seal, it was found that Professor L. P. Yandell was the author of the successful essay.

R. MARTIN, *Rec. Sec'y.*"

